# Biomass burning in the Amazon region causes DNA damage and cell death in human lung cells

**DOI:** 10.1038/s41598-017-11024-3

**Published:** 2017-09-07

**Authors:** Nilmara de Oliveira Alves, Alexandre Teixeira Vessoni, Annabel Quinet, Rodrigo Soares Fortunato, Gustavo Satoru Kajitani, Milena Simões Peixoto, Sandra de Souza Hacon, Paulo Artaxo, Paulo Saldiva, Carlos Frederico Martins Menck, Silvia Regina Batistuzzo de Medeiros

**Affiliations:** 1 Department of Pathology, School of Medicine, University of São Paulo, São Paulo, Brazil; 2 Department of Microbiology, Institute of Biomedical Sciences, University of São Paulo, São Paulo, Brazil; 3 Department of Medicine, Washington University in St. Louis, Saint Louis, Missouri, USA; 4 Department of Biochemistry and Molecular Biology, Saint Louis University School of Medicine, St. Louis, USA; 5 Institute of Biophysics Carlos Chagas Filho, Federal University of Rio de Janeiro, Rio de Janeiro, Brazil; 6 Federal University of Rio Grande do Norte, Biochemistry Department, Natal, Brazil; 7 National School of Public Health at Oswaldo Cruz Foundation, Rio de Janeiro, Brazil; 8 Institute of Physics, University of São Paulo, São Paulo, Brazil; 9 Cellular Biology and Genetics Department, Federal University of Rio Grande do Norte, Natal, Brazil

## Abstract

Most of the studies on air pollution focus on emissions from fossil fuel burning in urban centers. However, approximately half of the world's population is exposed to air pollution caused by biomass burning emissions. In the Brazilian Amazon population, over 10 million people are directly exposed to high levels of pollutants resulting from deforestation and agricultural fires. This work is the first study to present an integrated view of the effects of inhalable particles present in emissions of biomass burning. Exposing human lung cells to particulate matter smaller than 10 µm (PM_10_), significantly increased the level of reactive oxygen species (ROS), inflammatory cytokines, autophagy, and DNA damage. Continued PM_10_ exposure activated apoptosis and necrosis. Interestingly, retene, a polycyclic aromatic hydrocarbon present in PM_10_, is a potential compound for the effects of PM_10_, causing DNA damage and cell death. The PM_10_ concentrations observed during Amazon biomass burning were sufficient to induce severe adverse effects in human lung cells. Our study provides new data that will help elucidate the mechanism of PM_10_-mediated lung cancer development. In addition, the results of this study support the establishment of new guidelines for human health protection in regions strongly impacted by biomass burning.

## Introduction

Most of the overwhelming amount of research on exposure to air pollution is focused on urban centers and on the role of fossil fuels as the most important source of atmospheric pollutants. However, approximately 3 billion people in the world are exposed to air pollution from biomass burning, originating from using wood or coal as cooking fuel in simple stoves, home heating with open fires, deforestation, and agricultural practices^1^.

Biomass burning emits significant quantities of known pollutants hazardous to health, including several carcinogenic compounds^2^. World Health Organization (WHO) reported that in 2012, approximately 7 million people - one in eight total global deaths - as a result of exposure to air pollution^3^. Fire is a global phenomenon, and is an integral part of the earth’s ecosystem^4, 5^.

In particular, the Brazilian Amazon region contains world’s largest tropical forest and is considered, during the rainy season, one of the continental regions least affected by human activities^6, 7^. However, during the dry season, high concentrations of aerosol particles from biomass burning (mainly agricultural practices and deforestation) have been documented in this region^7, 8^. The combination of forest fires and human occupation has turned biomass burning into a serious public health threat. The majority of forest fires occur in the deforestation arc, a belt in the southern and western regions of the forest, directly impacting over 10 million people in the area^9^. Many studies in the area have identified severe effects on human health, such as increased incidences of asthma, morbidity and mortality, mainly in the most vulnerable populations such as children and elderly^10, 11^.

The smoke plume extends over millions of km^2^, covering large areas of South America, with significant health impacts extending far from the Amazon region^12, 13^. A recent study has estimated that reduction in the rate of deforestation in the Amazon in previous years has been preventing approximately 400 to 1,700 premature adult deaths annually, throughout South America^13^.

Studies show that inhabitants in the deforestation arc breathe air with high concentrations of particulate matter smaller than 10 µm (PM_10_). The problem is aggravated during the dry season, when high concentrations of PM_10_ have been measured (ranging from 400 up to 600 µg.m^−3^)^14^, exceeding the upper limits of concentration established by WHO (24 h exposure to PM_10_ – 50 µg.m^−3^) by 8 to 12 times. These inhalable particles have been classified as class 1 cancer-causing agents in 2013 by the International Agency for Research on Cancer (IARC)^15^. They can penetrate the alveolar regions of the lung, pass through the cell membrane, reach the blood and can accumulate in other human organs^16^.

Although epidemiological studies on the effects of urban PM on human health are numerous, there are relatively few that focused on the impact of air pollution resulting from biomass burning^2, 17^. Even scarcer are the studies that investigate the cellular and molecular mechanisms underlying PM toxicity. In one of these studies, Borgie and collaborators observed that PM increased the histone H2AX phosphorylation (γ-H2AX) (a DNA damage marker), telomerase activity, and induced epigenetic changes in bronchial epithelial cells^18^. Several groups reported that PM induces cell cycle alterations^19^, oxidative stress^20, 21^ and cell death^22^. Most of these studies focused on PM in urban areas. Recently, our group showed that organic PM_10_ in Amazon biomass burning emissions had mutagenic effect on plant cells and human lung cells^23, 24^. The objective of the present study was to investigate these effects in depth and provide a thorough analysis of the toxic cellular and molecular effects of relevant concentrations of PM_10_ resulting from Amazon biomass burning, on human lung. In fact, this manuscript is a sequel to an article published recently by our group characterizing in detail the chemical composition of the inhalable material whose health effects are investigated here^25^.

Our findings raise concern regarding human health of affected people, since we investigated the effects of PM on human lung cells using concentrations within plausible range of *in vitro* assays.

## Results

### PM_10_ from Amazon region reduces cell viability and increases ROS generation in human lung cells

**Figure 1:**
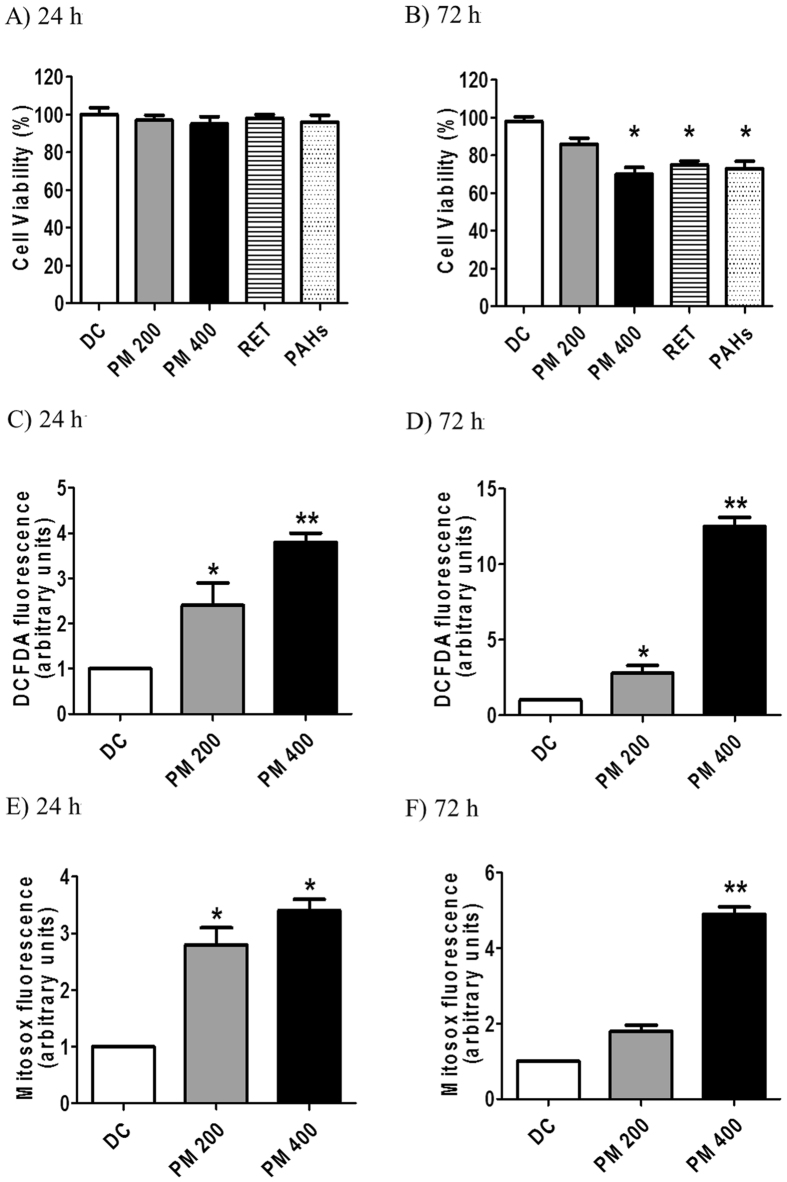
Cell viability (XTT) analysis of A549 human lung cells after 24 h (**A**) or 72 h (**B**) continuous treatment with organic PM_10_ (200 or 400 µg.mL^-1^). ROS induction under the same conditions was also evaluated, using the DCFDA (**C** and **D**) or the Mitosox (**E** and **F**) fluorescence probes. *p < 0.05 and **p < 0.01 are statistically significant compared to negative control (DMEM), according to Dunnett's test. DC: DMSO control (0.1%).

We first evaluated the viability of human lung epithelial cells (A549 cell line) exposed to 200 and 400 µg.mL^−1^ of organic PM_10_ (Fig. 1A). There was no significant difference in the viability of cells exposed to PM_10_ for 24 h compared to that of untreated cells (Fig. 1A). However, significant loss of viability was observed in cells exposed to 400 µg.mL^−1^ of PM_10_ for 72 h (Fig. 1B). We also evaluated the cytotoxicity of retene (RET) (Fig. 1A and B). The concentration of RET used in this study was 30 ng.mL^−1^, which corresponds to the contribution of RET to the organic PM extract at 400 µg.mL^−1^. Our data showed that treatment with this compound for 72 h was cytotoxic to human lung cells (Fig. 1B). Polycyclic aromatic hydrocarbons (PAHs) were used as positive control (10 ng.mL^−1^) for cytotoxicity analysis (Fig. 1A and B)^24^.

Since PM collected with different source characteristics was implicated in ROS generation^26, 27^, we investigated the ROS levels in A549 cells exposed to PM_10_. Intracellular ROS generation was significantly higher in cells exposed to PM_10_ for 24 and 72 h compared to the control cells (Fig. 1C and D). The effect was also dose and time-dependent. In particular, there was an 8.6-fold increase in fluorescence intensity in cells treated with PM_10_ for 72 h (Fig. 1D).

Mitochondrial superoxide indicator (MitoSOX) was used to evaluate the possible involvement of mitochondria in ROS formation. Flow cytometry analysis revealed that treatment with organic PM_10_ (200 and 400 μg.mL^−1^) markedly increased mitochondrial ROS generation in A549 cells in a dose and time-dependent manner, starting just after 24 h (Fig. 1E), and continuing till after exposure 72 h, specially at the higher concentration (Fig. 1F). These data suggest an important role of mitochondria in ROS generation upon exposure to PM_10_.

### PM_10_ induces the secretion of inflammatory cytokines by human lung cells

**Figure 2:**
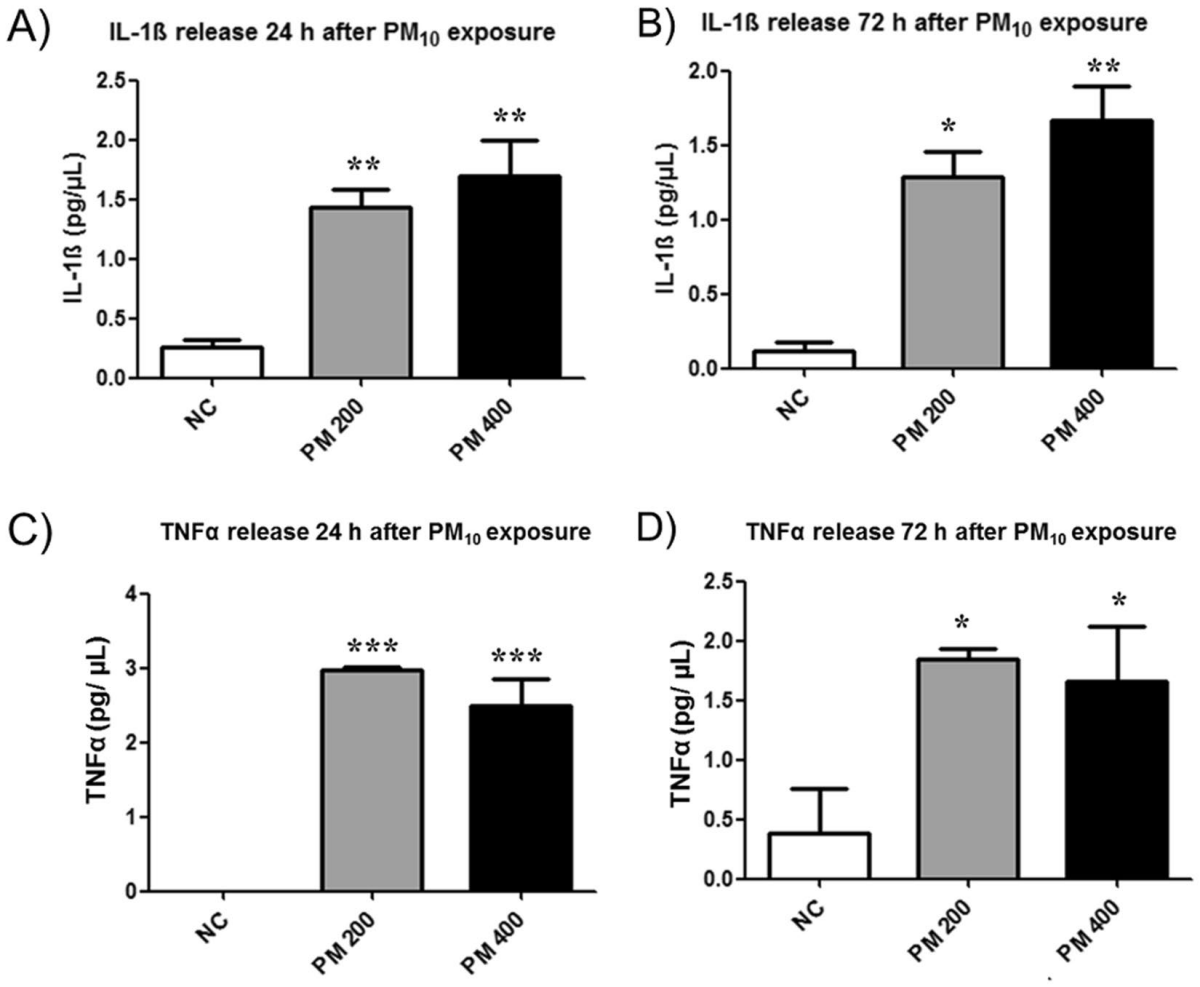
PM_10_ in biomass burning emissions induces the secretion of inflammatory cytokines by human lung cells. IL-1β release 24 h (**A**) and 72 h (**B**) after PM_10_ exposure (200 and 400 µg.mL^−1^); (**B**) TNF-α release 24 h (**C**) and 72 h (**D**) after PM_10_ exposure. *p < 0.05, **p < 0.01 and ***p < 0.001 statistically significant compared to negative control (NC) (DMEM), according to Bonferroni test.

Since PM_10_ known is to induce the secretion of inflammatory cytokines^28, 29^, we next investigated the concentrations of TNF-α and IL−1β in the culture media of cells exposed to PM_10._ IL-1β levels were significantly elevated in cells treated with 200 or 400 μg.mL^−1^ of PM_10_, for 24 or 72 h, in comparison to those in control cultures (Fig. 2A and B). Similar results were observed for TNF-α (Fig. 2C and D), indicating the induction of pro-inflammatory response in human lung cells exposed to PM_10_.

### PM_10_ induces autophagosome accumulation

**Figure 3:**
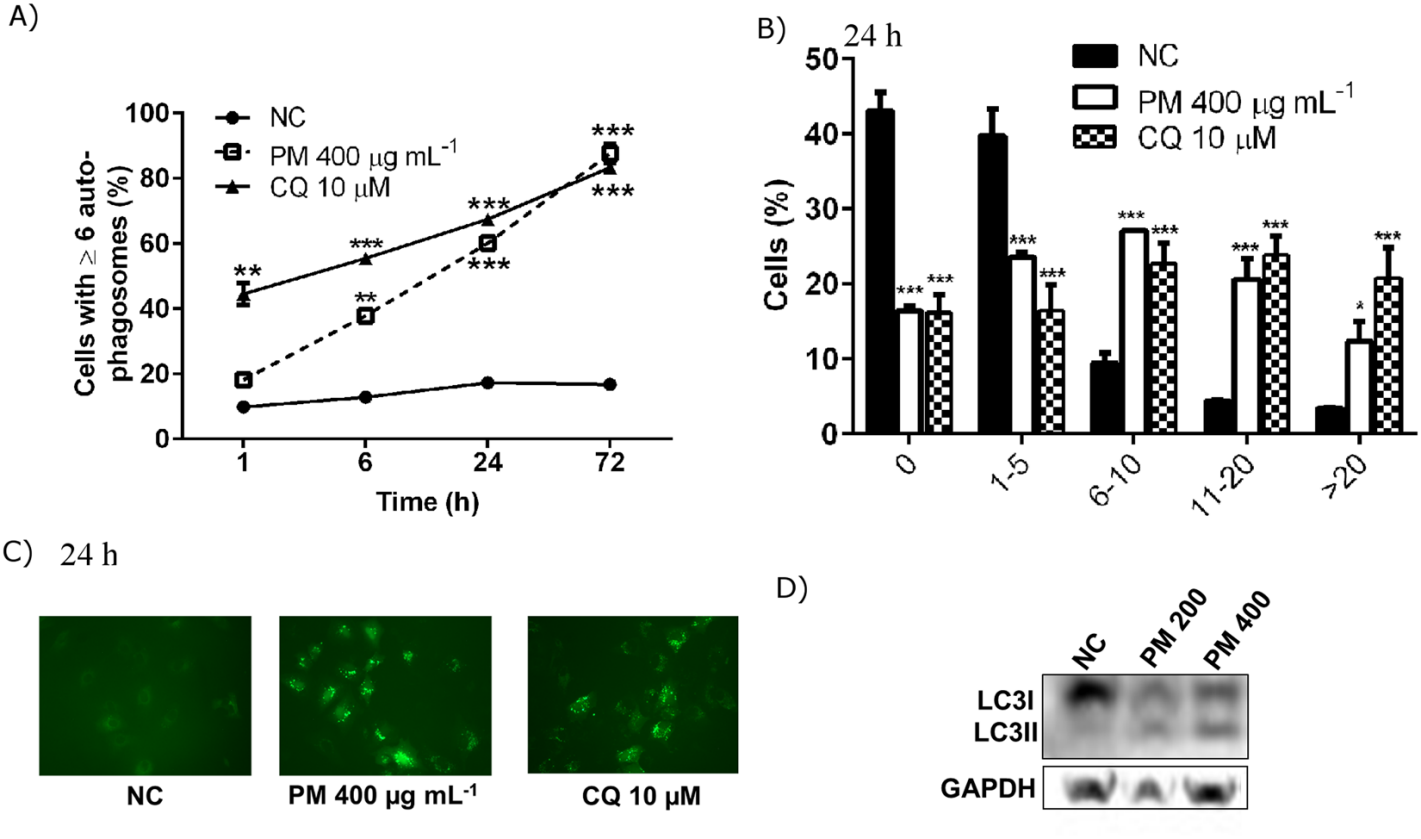
PM_10_ induces autophagosome accumulation in a time-dependent manner in human lung cells. (**A**) Quantification of autophagosome accumulation at 1, 6, 24 and 72 h after treatment with 400 μg.mL^−1^ of PM_10_. Chloroquine (CQ) 10µM was used as a positive control for autophagosome accumulation. (**B**) Comparison of number of autophagosome after 24 h exposure to PM_10_ or CQ_._ (**C**) Representative images of the formation of autophagosome in human lung cells with negative control (NC), 400 μg.mL^−1^ of PM_10_ or CQ (10µM). (**D**) Expression levels of LC3I and LC3-II as measured by Western blotting. The blots were cropped. *p < 0.05, **p < 0.01 and ***p < 0.001 statistically significant compared to NC according to Bonferroni test.

The increase in ROS generation prompted us to investigate autophagy^30^, a pathway that is induced by ROS and also can mitigate the generation of ROS. For investigating the autophagy response, we chose 400 μg.mL^−1^ of PM_10_ concentration, since this dose caused a more dramatic increase in ROS levels after 72 h treatment (Fig. 1D and F). As shown in Fig. 3A,B and C, the number of A549 cells engaged in autophagy (as measured by the formation of cytoplasmic, fluorescent round-shape GFP-LC3 structures) increased after exposure to 400 μg.mL^−1^ of PM_10_ in a time-dependent manner.

To confirm these results, we measured, by western blotting, the conversion of unprocessed cytosolic LC3-I to the LC3-II (autophagosome-associated) form, following PM_10_ treatment. Our results show an increase in LC3-II in A549 cells exposed to 200 and 400 μg.mL^−1^ of PM_10_ for 24 h, demonstrating LC3-processing (Fig. 3D). These results confirmed that PM_10_ exposure induced autophagy in A549 cells.

### PM_10_ induces DNA damage and cell cycle alterations in human lung cells

**Figure 4:**
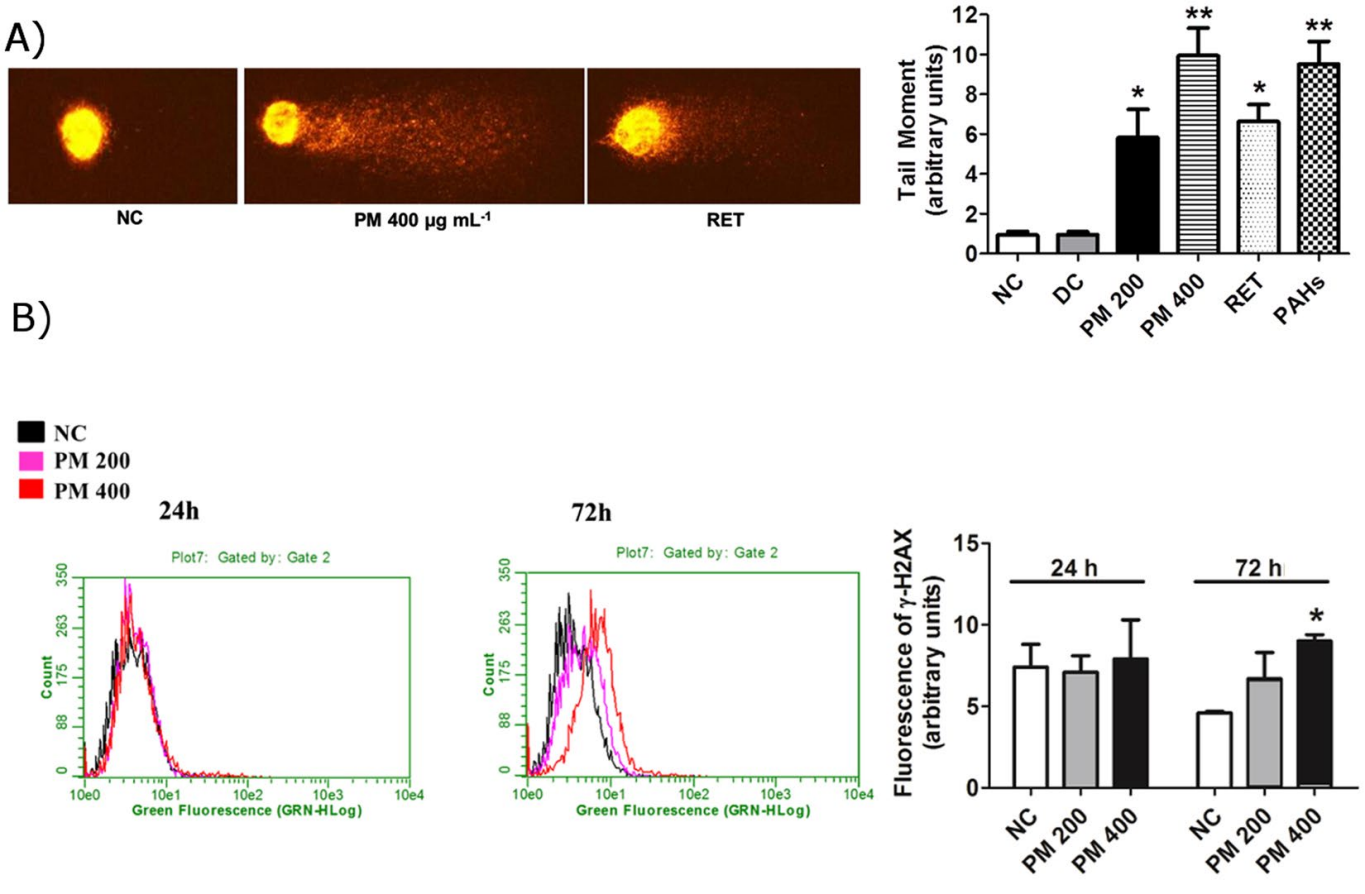
PM_10_ from biomass burning emissions induces DNA damage in human lung cells. (**A**) Image of comet test after treating A549 human lung cells with PM_10_; mean of comet tail moment after treatment with 200 and 400 μg.mL^−1^ of PM_10,_ RET (30 ng. mL^−1^) and PAHs mix (10 ng.mL^−1^) for 24 h. (**B**) γ-H2AX formation after 24 and 72 h treatment with PM_10_. *p < 0.05 and **p < 0.01 statistically significant compared to negative control (NC) (DMEM) according to Dunnett's test. DC: DMSO control (0.1%).

An increase in ROS levels can result in DNA damage and mutagenesis^31^. Therefore, we evaluated the induction of DNA lesions using comet assay. Analysis was performed 24 h after treatment with PM_10_ since, at later time points, DNA fragmentation induced by cell death could interfere with the results. Results of this analysis showed that organic PM_10_ induced significant DNA damage, as evidenced by tail formation at all doses tested. We used the PAHs mix as positive control in this experiment and confirmed its genotoxic effect (Fig. 4A). We also assessed the genotoxic potential of RET, since there are very few studies investigating the genetic damage caused by this compound generated during biomass combustion. RET induced DNA damage in human lung cells, and the difference in the extent of damage between treatment group and negative control was significant (Fig. 4A).

Additionally, we evaluated the formation of γ-H2AX, a widely used DNA damage marker^32^. The nuclei of human lung cells exposed to PM_10_ for 24 h stained positive for γ-H2AX, although the degree of staining was not significant. The number of γ-H2AX-positive cells increased in a time and dose-dependent manner, and a significant increase in **γ-**H2AX formation in A549 cells was observed at higher concentration of PM_10_ (400 µg.mL^−1^) after 72 h treatment (Fig. 4B).

**Figure 5:**
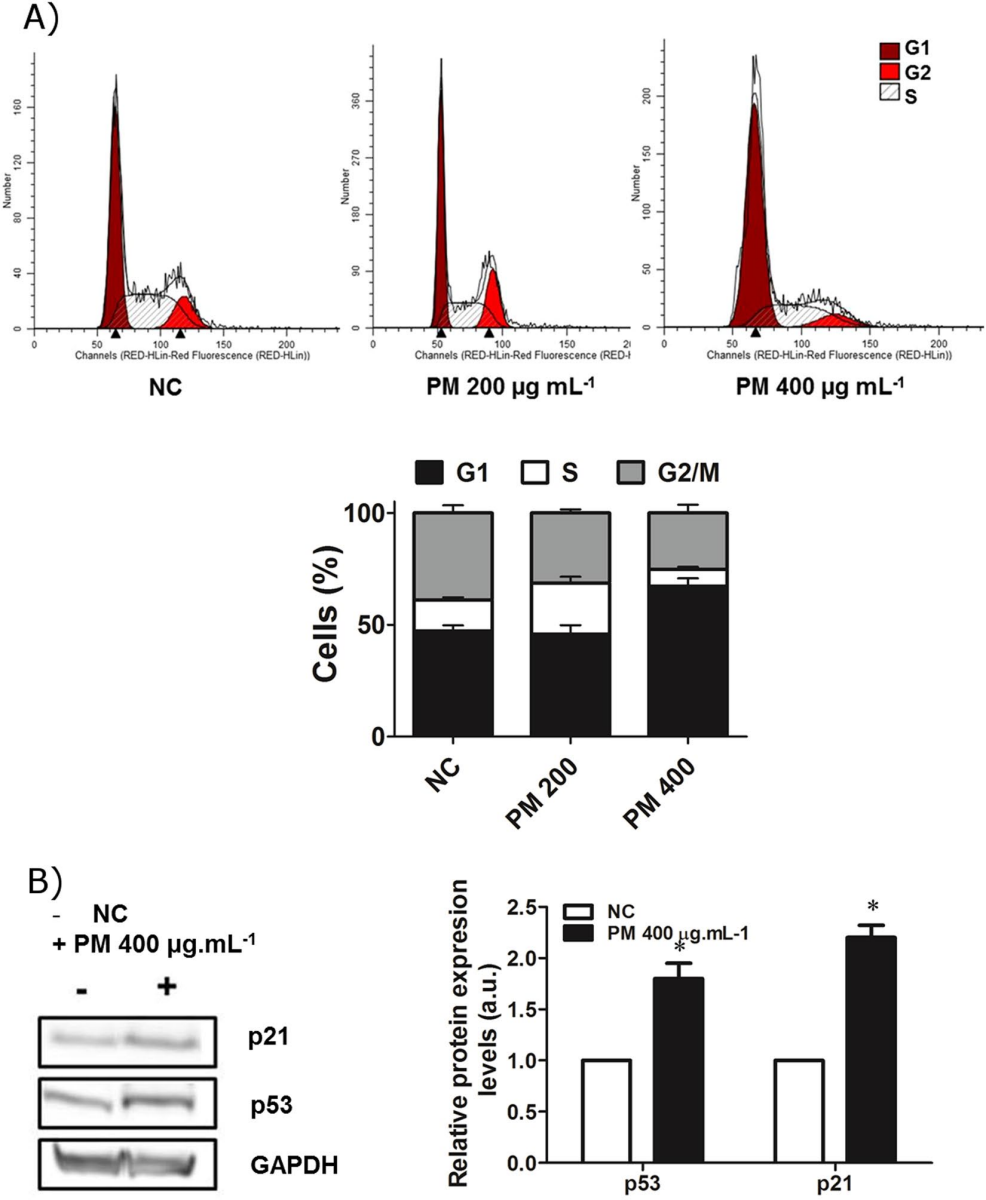
Organic PM_10_ from Amazon region induces the expression of p53 and p21 and alters cell cycle. (**A**) Cell cycle profile of the A549 cells treated with organic PM_10_ for 24 h. (**B**) Expression of p53 and p21 proteins in cells treated with organic PM_10_ for 24 h. Quantification from three independent experiments is shown. Western blots were cropped. *p < 0.05 statistically significant compared to negative control (NC) according to Dunnett’s test.

We then investigated whether these inhalable particles can alter the cell cycle of human lung cells. Figure 5A shows the cell cycle profile of the A549 cells exposed to organic PM_10_ for 24 h. The results showed an increase in the number of cells in G1 phase, although only in cells treated with 400 μg.mL^−1^ of PM_10_, consistent with the induction of **γ-**H2AX formation at this dose.

The mechanism underlying the cell cycle alterations was investigated by analyzing the expression of two key proteins, p53 and p21, by western blotting. There was an increase in the levels of these proteins in cells treated with organic PM_10_ for 24 h (Fig. 5B), suggesting that DNA damage induced by PM_10_ leads to cell cycle alterations.

### PM_10_ induces apoptosis and necrosis in human lung cells

We, next investigated if PM_10_ can induce cell death in human lung cells, possibly as a consequence of DNA damage. For these assays, we chose the highest concentration of organic PM_10_ (400 µg.mL^−1^) used in this study.

**Figure 6:**
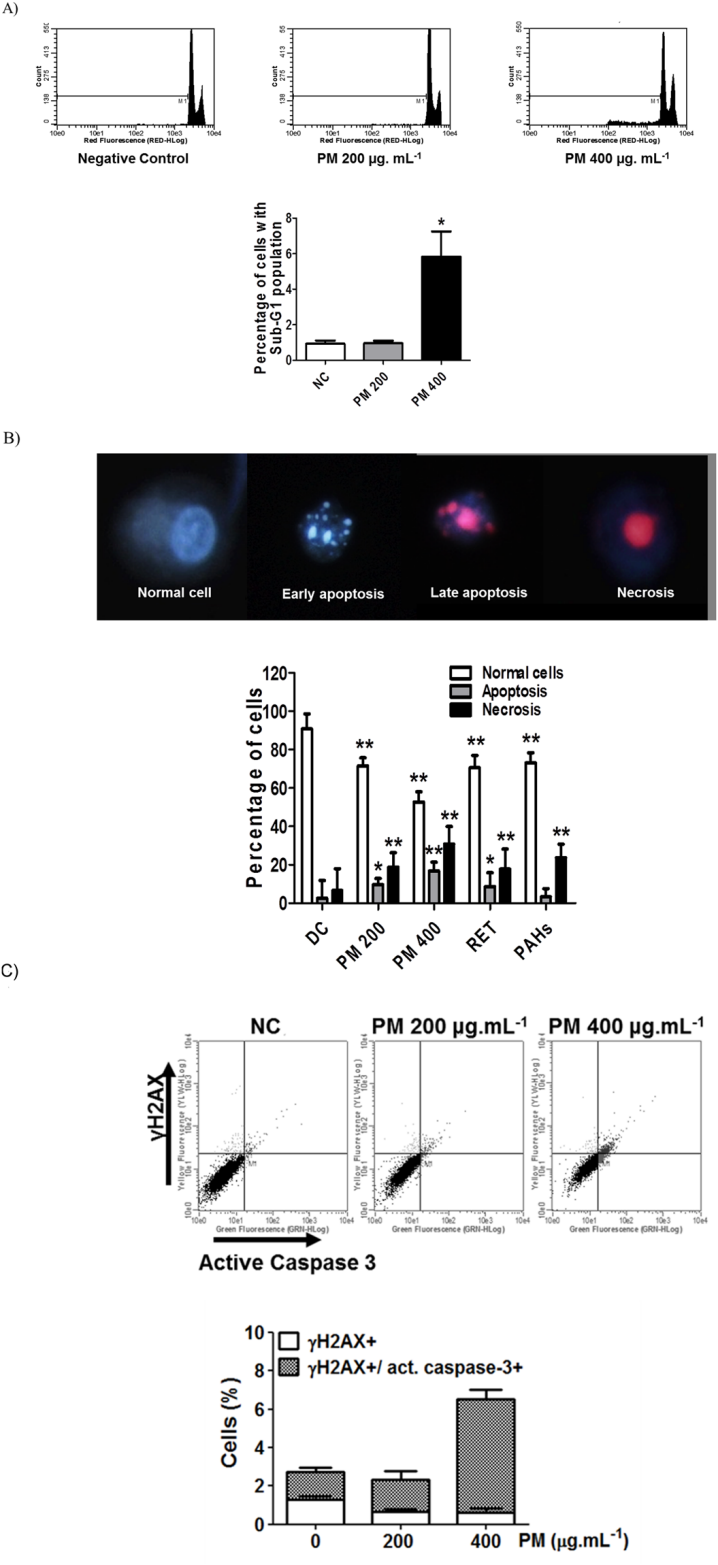
PM_10_ from biomass burning emissions induces cell death. (**A**) Nuclear fragmentation in human lung cells after treating the cells with the organic fraction of PM_10_ for 72 h. (**B**) Cell death assay by fluorescence microscopy that distinguishes apoptosis (early and late) from necrosis. (**C**) γ-H2AX and active caspase-3 analyses by flow cytometry after 72 h exposure to PM_10_. NC: negative control; DC: DMSO control (0.1%) *p < 0.05 and **p < 0.01 statistically significant compared to NC according to Dunnett’s test.

To evaluate the cell death response precisely, we quantified the sub-G1 cell population, which represents cells with fragmented nuclei, an indicator of apoptosis induction^33^. After 72 h exposure to organic PM_10_, we detected an increase in sub-G1 cells (Fig. 6A). To complement this analysis, we stained the cells with PI and Hoescht, and observed nuclear morphology, as well as PI uptake, using a fluorescence microscope. Figure 6B shows the occurrence of early (6.8%) and late apoptosis (10%), as well as necrosis (30.8%) after 72 h continuous treatment with PM_10_. Interesting, the RET induced cell death mainly by necrosis in human lung cells with a significant difference compared to negative control.

Based on these results, we hypothesized that the DNA fragmentation that occurs during apoptosis increases the levels of **γ-**H2AX staining. Therefore, we analyzed the active caspase-3 content, concomitantly with **γ-**H2AX staining and observed a clear association between these two markers (Fig. 6C). To validate these results, we performed this assay with ultraviolet-C (UVC) light, which induces two types of **γ-**H2AX staining, moderate and high. Only the latter correlates with cell death^34^. Strikingly, PM-induced **γ-**H2AX differs from that induced by UVC, and is exclusively related to apoptosis (Figure S1).

## Discussion

It is well recognized that PM from urban air pollution induces lung cancer^15^. However, the exact mechanisms underlying this effect are still unclear. The present study revealed that the PM_10_ emitted during biomass burning in the Amazon region caused toxic effects at molecular and cellular levels in human lung cells. Here, we present for the first time, an integrated analysis of the effects of particulate emissions from the forest fires of Amazonian deforestation arc, on oxidative stress, inflammatory response, autophagy induction, cell cycle arrest, DNA damage, and cell death of human lung cells. It has been proposed that these pathways represent important toxicological mechanisms associated with PM-induced lung cancer^35, 36^.

It is clear that the effects of PM depend, not only on the particle size, but also on the composition of PM. The identification of all the toxic components within the PM ensemble is an extremely challenging task, given the complexity of the composition and number of compounds^37^. PAHs are known to be largely responsible for the toxicity of PM generated from a variety of sources^15, 38^. An earlier study quantified the PAH content in the organic extract used in this study, identifying that 40% of these compounds were known carcinogens^25^. One key compound identified in this work was dibenzo[a,h]anthracene. Another study of our group, focusing on nitrated and oxygenated PAHs (de Oliveira Galvão, manuscript accepted)^39^, also established the contribution of nitrated and oxygenated-PAHs to human carcinogenic risk from the same material, indicating the identity of the components responsible for the effects studied here.

We investigated *in vitro*, the effects of PM_10_ on human lung cells using concentrations within the plausible range. Our results show that ROS generation occurs as an early event. We hypothesized that ROS-dependent oxidative stress caused by PM_10_ would lead to the release of inflammatory cytokines. Consistent with the results of previous studies, organic fractions from urban emissions^40, 41^ and wildfire emissions^42, 43^ strongly induced the secretion of IL-1β and TNF-α. In addition, the impact of long-range transport episodes of wildfire smoke was investigated in the mouse cell line, RAW 264.7. The cytokine production observed in this study^44^ was substantially lower, compared to that induced by corresponding particles of the seasonal average period. It is suggested that this reduction could be due to the chemical transformation of the organic fraction during aging^44^. Besides, it is interesting to note that 1-nitropyrene, a compound identified in the PM extract used here (de Oliveira Galvão, manuscript accepted)^39^, induced oxidative DNA damage by generation of ROS in A549 cells^45^, which may explain the previously identified mutagenic and carcinogenic potential of this compound^46^.

Moreover, mitochondria are also responsible for the increase in ROS, as evidenced by the formation of significant quantities of mitochondrial superoxide after exposure to PM_10_. An earlier study showed the involvement of mitochondria in ROS production in rat alveolar type II and human lung cells exposed to urban PM^47^. Interestingly, in another study using urban pollution samples, low dose PM increased ROS production in bronchial epithelial cells, but not the formation of mitochondrial superoxide. Thus, besides superoxide, those other mitochondria-independent pathways may also drive ROS generation in certain cell types^19^.

Several studies have reported that polycyclic aromatic quinones cause severe adverse biological effects such as allergic inflammation via induction of the ROS pathway^48–50^. Shang *et al*.^26^, for example, showed that some quinones (1,2-naphthoquinone, 2-methylanthraquinone, 9,10-phenanthrenequinone, 2-methyl-1,4-naphthoquinone, and acenaphthenequinone) caused viability decrease, cellular LDH release, DNA damage and ROS production in A549 cells. Correspondingly, 2-methylanthraquinone was the most abundant compound among oxygenated-PAHs in our samples (de Oliveira Galvão, manuscript accepted)^39^. Therefore, it is likely that this compound also contributed significantly, because of its high redox potency, to the adverse health effects reported here.

Furthermore, excessive levels of ROS can cause severe damage to DNA and proteins. In this respect, autophagy has been conventionally considered a pathway that is activated in response to these insults, to maintain cellular homeostasis^51^. Our results clearly showed the accumulation of autophagosomes in PM_10_-treated cells. In agreement, Deng and collaborators showed that PM-induced oxidative stress probably plays a key role in autophagy induction in A549 cells (same cellular model used in this work), which may contribute to PM-induced impairment of pulmonary function^52^. These authors also reported that PM could elicit oxidative stress by decreasing the activity of antioxidant enzymes activity, finally leading to cell death^52^.

Another interesting finding of this study was the cell cycle arrest at G1-phase and formation of DNA strand breaks in response to PM_10_ treatment. In this context, Longhin and collaborators showed that PM altered cell cycle significantly in the human epithelial cells just after 3 h of exposure^19^. DNA damage has also been reported to result from exposure to PM in coke oven^53^, vehicular^54^, and industrial emissions^55^. Different mechanisms have been proposed to explain these effects, such as DNA strand breaks caused by the oxidative stress exerted by different agents (including PAHs)^56^ and formation of bulky DNA adducts by the direct action of reactive PAHs^57^.

An appropriate response of cells to DNA damage involves activation of checkpoint proteins that regulate cell cycle progression. In fact, we noticed an increase in the expression of p53 and its downstream target, p21, which is capable of inducing cell cycle arrest. This is an early event after PM_10_ treatment. Similarly, a recent study reported that urban PM induced G1 arrest in alveolar epithelial cells with p21 playing a critical role in the regulation of cell cycle and preventing apoptosis^58^. These effects have been observed when the cells were exposed to carcinogenic PAHs. For example, dibenzo[a,l]pyrene and benzo[a]pyrene are able to induce the formation of DNA adducts, increases in the levels of p53 and p21, and G1 or G2/M arrests in human mammary carcinoma cells (MCF-7)^59^.

These early responses are not sufficient to counteract the DNA damage effects, if the exposure to PM_10_ continues, which will lead to the activation of cell death processes. Our results showed cell death by apoptosis and necrosis after 72 h of exposure to PM_10_. Interestingly, we observed a positive correlation between the activation of caspase-3, an apoptosis protease effector, and γ-H2AX formation, probably due to apoptotic DNA fragmentation^60^. A previous study also reported that urban aerosols during a biomass burning episode in Indonesia caused cell death in A549 cells and that this effect could be related to the cytotoxic effects of metals and PAHs contained in the ambient PM^61^.

Real-world biomass burning emissions contain myriad compounds, including those recognized as carcinogenic and toxic. These compounds were also present in the PM samples used in this study. Nonetheless, there are many other compounds whose health effects have not yet been studied. For example, RET is a PAH normally associated with wood combustion^62^ and was present in high concentrations in the samples used in this study^25^. RET has not been included in the risk assessment of PAHs by US-EPA^63^. Given its high concentrations in the Amazon samples, we decided to investigate its genotoxic effect. We found that it is highly genotoxic, and also caused cell death by necrosis. These results provide new evidence for the effect of RET on human health, especially in regions impacted by biomass burning. Further studies focusing on the role of RET in lung cancer development are therefore strongly encouraged.

**Figure 7:**
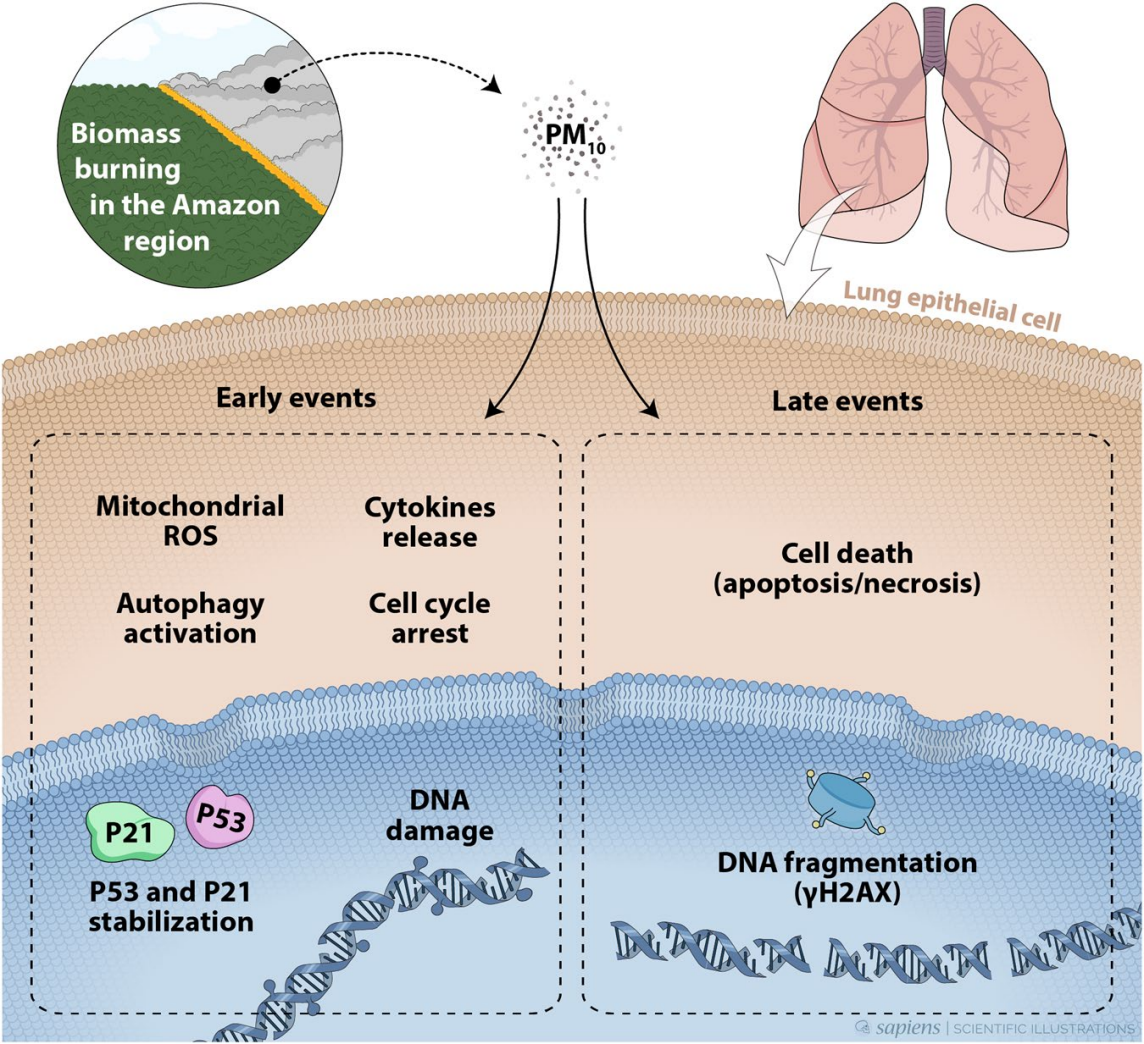
Proposed mechanism of action of PM_10_ from biomass burning emissions in the Amazon region. As early events, formation of DNA strand breaks, increased production of ROS and cytokines, mitochondrial alterations, and autophagy could be observed clearly. PM_10_ also caused G1 cell cycle arrest and an increase in the expression of p53 and p21 proteins. In the event of continued exposure, this early response is not sufficient to counteract the effects of PM_10_. During late events, both apoptosis and necrosis are activated. DNA fragmentation induces γ-H2AX formation. The genetic instability driving tumorigenesis is fueled by DNA damage that can cause molecular alterations (e.g., mutations) and can play an important role in lung cancer development.

Considering all these data together, we propose a mechanism of action of PM_10_ present in the emissions from biomass burning in the Amazon region, in human lung cells (Fig. 7). DNA damage is an early molecular hallmark of exposure to PM. Organic matter present in PM_10_ extracts can transpose human lung cells and generate oxidative stress by mitochondrial ROS generation. ROS induces cytokine release contributing to the oxidative stress, leading to DNA damage. This situation can activate autophagy for reestablishing normal cellular homeostasis as well as causing cell cycle arrest for dealing with this damage. If the cells are not able to counteract these effects, cell death pathways are activated, increasing DNA fragmentation by apoptosis and also necrosis. DNA fragmentation also induces the formation of γ-H2AX. The resultant loss of cells may explain some acute effects of respiratory diseases. However, if the cells are not able to deal with DNA damage and not progress to cell death, mutagenesis can occur leading to the development of lung cancer^64^.

To summarize, this study investigated the acknowledged adverse health effects of deforestation fires in the Amazon Basin, using several analytical techniques. The PM in the emissions from biomass burning in the Amazon region has been observed to cause DNA damage and cell death. The results described here could be used for detailed chemical speciation, which will provide clues to compounds involved in the negative health effects. Our study provides new data on the mechanism of action of PM_10_ in contributing to the development of lung cancer. A better understanding of the pathways by which particulate pollutants contribute to the burden of lung diseases, is an important step in reducing the risk of adverse respiratory effects resulting from exposure to biomass burning emissions and to develop individual and community-level interventions. It is also an important contribution to the establishment of new guidelines for human health protection in regions strongly impacted by biomass burning.

## Methods

### Particle collection and PM_10_ sample preparation

The aerosol sampling was conducted in the Brazilian Amazon region, located about 5 km north of Porto Velho. A total of 19 PM_10_ samples were collected with quartz fiber filters using a high-volume sampler during the dry season (August–October/2011). The samples were extracted as previously described^25^. The organic extract was dissolved in dimethyl sulfoxide (DMSO) and was used for cell treatment.

### Cell culture, PM_10_ treatment and cell proliferation assay

A549 cells were routinely grown at 37 °C, in a humidified 5% CO_2_ in Dulbecco’s Modified Eagle’s Medium (DMEM) containing phenol red and supplemented with 10% fetal bovine serum (FBS) and antibiotics (0.1 mg.mL^−1^ penicillin, 0.1 mg.mL^−1^ streptomycin and 0.25 mg.mL^−1^ fungizone).

Human lung cells were exposed to organic PM_10_ (200 µg.mL^−1^ and 400 µg.mL^−1^). It is important to note that, despite allowing detailed analysis, *in vitro* cell exposure studies require pre-treatment of inhalable material such as liquid extraction and thus could limit/modify the effects of real-world exposure.

The exposure doses to be used in the present study were difficult to determine, since there were no previous studies on the *in vitro* mechanisms of particles emitted during the Amazon fires. We used as reference, the estimated alveolar mass deposition accumulating over 24 h in human adults exposed to air concentrations identified in the Amazon region. PM_10_ concentrations used for mass calculation were 60 µg.m^−3^ (peak identified during forest fire in Porto Velho - 2011) and 118 µg.m^−3^ (peak identified during forest fire in Porto Velho - 2010)^7^. The average daily inhalation rate used in this study was 16 m^3^.day^−1^ for adults^65^ and the alveolar deposition rate of PM_10_ in the human respiratory tract is approximately 20%^66^. It was, of course, not possible to directly extrapolate the real-world conditions, since we did not correct the dose with respect to alveolar surface. In addition, we used liquid extracts, which eliminated the effect of surface/reactivity properties of small particles. However, we consider reasonable to say that our concentrations were within a plausible range.

RET, the most abundant PAH in Amazon samples, was also evaluated in this study. A mix of PAHs (acenaphthylene, phenanthrene, anthracene, fluorene, pyrene, benz[a]anthracene, chrysene, benzo[b]fluoranthene, benzo[k]fluoranthene, benzo[a]pyrene, indene[1,2,3-c,d]pyrene, dibenz[a,h]anthracene and benzo[g,h,i]pyrene; EPA 525 PAH Mix A (48953-U, Sigma)) was also used as positive control based on previous studies^24, 67^. Cell viability was performed 24 and 72 h after PM_10_ treatment using Cell Proliferation Kit II (XTT) (Roche, Basel, Switzerland) as described elsewhere^68^. The absorbance was measured at 492 nm (650 nm as reference). Cell viability was expressed as percentage of control cells.

### Alkaline comet assay

DNA strand breaks were detected using alkaline comet assay described in an earlier study^34^. A549 cells exposed to PM_10_ were trypsinized and resuspended in 180 µL 0.5% low melting point agarose at 37 °C. The cells were then spread homogenously on two microscope slides pre-coated with 1.5% agarose, immediately covered with coverslips and kept at 4 °C for 10 min. Cells were then lysed overnight in chilled lysis solution at 4 °C. The slides were then placed horizontally in an electrophoresis chamber with cold alkaline buffer (300 mM NaOH, 1 mM EDTA, pH > 13) for 25 min at 25 V and 300 mA. The slides were then neutralized (0.4 M Tris, pH 7.5) and fixed with ice-cold 100% ethanol. Finally, the slides were stained with ethidium bromide and imaged with a fluorescence microscope (Axiovert 200, Zeiss) at the magnification of 400x. At least 100 comets per slide were scored, on duplicate slides, using the Komet 6.0 software. The results were plotted by tail moment.

### ROS formation

ROS formation were determined using DCFH-DA (2′,7′-dichlorofluorescein diacetate, Sigma-Aldrich,35845). After exposure to PM_10_, A549 cells were detached with trypsin and incubated with DCFHA-DA dye for 30 min at 37 °C in DMEM without phenol red and supplemented with 0.2% FBS. The green fluorescence intensity in each well was quantified using flow cytometry (Guava® easyCyte, USA). Results are shown in relative fluorescence units.

### Mitochondrial superoxide formation

Mitochondrial superoxide production was measured by flow cytometry following staining with MitoSOX Red mitochondrial superoxide probe (Life Technologies, M36008). A549 cells treated with PM_10_ were detached by trypsin digestion. The cells were resuspended in DMEM without phenol red and supplemented with 0.2% FBS and incubated with 5 µM MitoSOX reagent for 20 min at 37 °C in the dark. The cells were washed twice with PBS and assessed using flow cytometry. Results are shown as relative fluorescence units.

### Cytokines release analysis

In order to evaluate the release of pro-inflammatory cytokines (IL-1β and TNF-α), we exposed A549 cells to organic PM_10._ The supernatant was snap frozen and stored at −80 °C until analysis by Cytometric Bead Array assay, using the Human Inflammatory Cytokines Kit (BD Biosciences, USA). We used the BD Accuri C6 (BD Biosciences) flow cytometer along with the FCAP 3.0 software to analyze the fluorescence of each bead in the FL2 and FL3 channels, which correspond to the concentration of the different cytokines.

### Analysis of autophagosome formation using GFP-LC3-expressing cells

Autophagosome formation, a hallmark of autophagy^69^, was monitored to establish the dynamics of autophagic process in PM_10_-exposed cells. For this purpose, we generated A549 cells stably expressing LC3 (a critical component of autophagosome formation) fused to GFP (GFP-LC3), and observed autophagosome formation under a fluorescence microscope, as described elsewhere^69^. Chloroquine treatment was used as a positive control for autophagosome visualization^33^.

### Sub-G1 and cell cycle analysis by flow cytometry

Sub-G1 and cell cycle analysis were performed as previously described^70^. A549 cells treated with PM_10_ were trypsinized, washed with PBS, and fixed with chilled 70% ethanol for at least 24 h, at −20 °C. Staining was performed at room temperature for 30 min in filtered PBS containing 200 µL propidium iodide (PI) solution (200 mg. mL^−1^ RNase A, 20 mg. mL^−1^ PI, and 0.1% Triton X-100 in PBS) and each sample was analyzed in a flow cytometer. Guava Express Plus software was used to quantify sub-G1, and cell cycle analysis was performed using ModFit LT software.

### Double staining of γ-H2AX and active caspase-3 by FACS

For concomitant analyses of γ-H2AX and active caspase-3 by FACS, the protocol was adapted from Quinet *et al*.^34^. Briefly, adherent cells were combined and fixed with 1% formaldehyde in ice, washed in PBS, and stored in 70% ethanol for at least overnight at −20 °C. Cells were blocked and permeabilized with BSA-T buffer (0.2% Triton X-100, 1% BSA in PBS). For γ-H2AX staining, samples were incubated with 1/500 anti-γ-H2AX antibody (05–636 Millipore) for 3 h at room temperature followed by two washes with BSA-T buffer and incubation with 1/200 anti-mouse TRITC (tetramethylrhodamine) secondary antibody (T5393 Sigma-Aldrich) for 1 h at room temperature. After washing twice with BSA-T buffer, samples were incubated with 1/50 FITC- (fluorescein iso-thiocyanate) conjugated anti-active caspase-3 antibody (559341 BD Pharmigen) overnight at 4 °C. Fluorescence spectral overlap was compensated using positive controls for both γ-H2AX or active caspase-3 staining. Data was acquired on a Guava Flow Cytometer and analyzed using CytoSoft Data Acquisition and Analysis Software (Millipore).

### Assessment of cell death

Cell death assessment was performed as described^71^. Briefly, A549 cells were collected by trypsinization and resuspended in 200 μL PBS. Ten microliters of this suspension was incubated for 5 min with 2 μL dye mixture containing 1 mg.mL^−1^ PI (Sigma-Aldrich), 1.5 mg.mL^−1^ diacetate fluorescein (Invitrogen, Life Technologies), and 1 mg.mL^−1^ Hoechst 33342 (Invitrogen, Life Technologies) at 37 °C, and transferred to a microscope slide. For each treatment, the presence of live cells, early apoptosis, late apoptosis, and necrosis were evaluated in 500 cells (in triplicate) using a Zeiss microscope Bloom. Healthy cells and early apoptotic cells are refractory to PI staining, and show intact or fragmented nuclei, respectively, in blue (Hoechst staining). Late apoptotic cells and necrotic cells display fragmented or intact nuclei, respectively and stain red, since they are permeable to PI.

### Protein extraction, quantification, and western blot analysis

Total protein extracts were analyzed as previously described^70^. Quantification was performed using the Pierce BCA Protein Assay kit (Thermo Scientific, USA). Fifty micrograms of total protein from cell extracts was separated on a 10% SDS polyacrylamide gel, and blotted onto a nitrocellulose membrane. Membranes were blocked for 1 h in 5% (w/v) milk powder in PBS-Tween 20 (PBST), incubated overnight at 4 °C with primary antibody, washed three times with PBST, and incubated for 1 h with secondary antibody. After final washes with PBST, the blots were developed using an enhanced chemiluminescence detection system (Immobilon Western, Merck Millipore). Protein expression was detected using anti-p53 (1:500, M7001, Dako Denmark A/S); anti-p21 (1:200, sc-56335, Santa Cruz, Santa Cruz, CA, USA); anti-LC3 (1:1000 dilution, L8918, Sigma-Aldrich); anti-GAPDH (1:1000 dilution, Santa Cruz, Santa Cruz, CA, USA) antibodies.

### Statistical analysis

Statistical analysis was performed using the Statistical Package for Social Sciences (SPSS) version 15.0 and Prism 5, GraphPad Software Inc. Statistical significance was assessed using one-way ANOVA followed by the Dunnet’s test or Bonferroni test. The mean differences were considered significant at p ≤ 0.05.

## Electronic supplementary material

Supplementary Information
