# Supplementary material for: Biomass burning in the Amazon region causes DNA damage and cell death in human lung cells
Source: Sci Rep. 2017 Sep 7;7:10937. doi: 10.1038/s41598-017-11024-3 (PMC5589902; doi:10.1038/s41598-017-11024-3)
Supplement: Supplementary file 1 — Supplementary Information [file 41598_2017_11024_MOESM1_ESM.doc]

**Biomass burning in the Amazon region causes DNA damage and cell death in human lung cells**

Nilmara de Oliveira Alves^1*^, Alexandre Teixeira Vessoni^2,3^, Annabel Quinet^2,4^, Rodrigo Soares Fortunato^5^, Gustavo Satoru Kajitani^2^, Milena Simões Peixoto^6^, Sandra de Souza Hacon^7^, Paulo Artaxo^8^, Paulo Saldiva^1^, Carlos Frederico Martins Menck^2^ and Silvia Regina Batistuzzo de Medeiros^9^

^1^ Department of Pathology, School of Medicine, University of São Paulo, São Paulo, Brazil;

^2^ Department of Microbiology, Institute of Biomedical Sciences, University of São Paulo, São Paulo, Brazil;

^3^ Department of Medicine, Washington University in St. Louis, Saint. Louis, Missouri, USA;

^4^ Department of Biochemistry and Molecular Biology, Saint Louis University School of Medicine, St. Louis, USA;

^5^Institute of Biophysics Carlos Chagas Filho, Federal University of Rio de Janeiro, Rio de Janeiro, Brazil;

^6^ Federal University of Rio Grande do Norte, Biochemistry Department, Natal, Brazil;

^7^ National School of Public Health at Oswaldo Cruz Foundation, Rio de Janeiro, Brazil;

^8^ Institute of Physics, University of São Paulo, São Paulo, Brazil;

^9^ Cellular Biology and Genetics Department, Federal University of Rio Grande do Norte, Natal, Brazil.

ATV: alevessoni@gmail.com; AQ: annabel.quinet@gmail.com; RSF: [rodrigof@biof.ufrj.br](mailto:rodrigof@biof.ufrj.br); GSK: [gustkajitani@gmail.com](mailto:gustkajitani@gmail.com); MSP: [milena.simoes.peixoto@gmail.com](mailto:milena.simoes.peixoto@gmail.com); SSH: sandrahacon@gmail.com; PA: [artaxo@if.usp.br](mailto:artaxo@if.usp.br); PS: [pepino@usp.br](mailto:pepino@usp.br); CFMM: [cfmmenck@usp.br](mailto:cfmmenck@usp.br); SRBM: [sbatistu@gmail.com](mailto:sbatistu@gmail.com).

***Correspondence should be addressed to:**NOA: [nilmaraoalves@gmail.com](mailto:nilmaraoalves@gmail.com).

School of Medicine of University of São Paulo, Department of Pathology, Laboratory of Experimental Air Pollution (LIM05) ZIP CODE 01246-903, São Paulo, SP, Brazil

Tel. # 55.11.3061-8531; Fax # 55.11.3061-725

Supplementary information


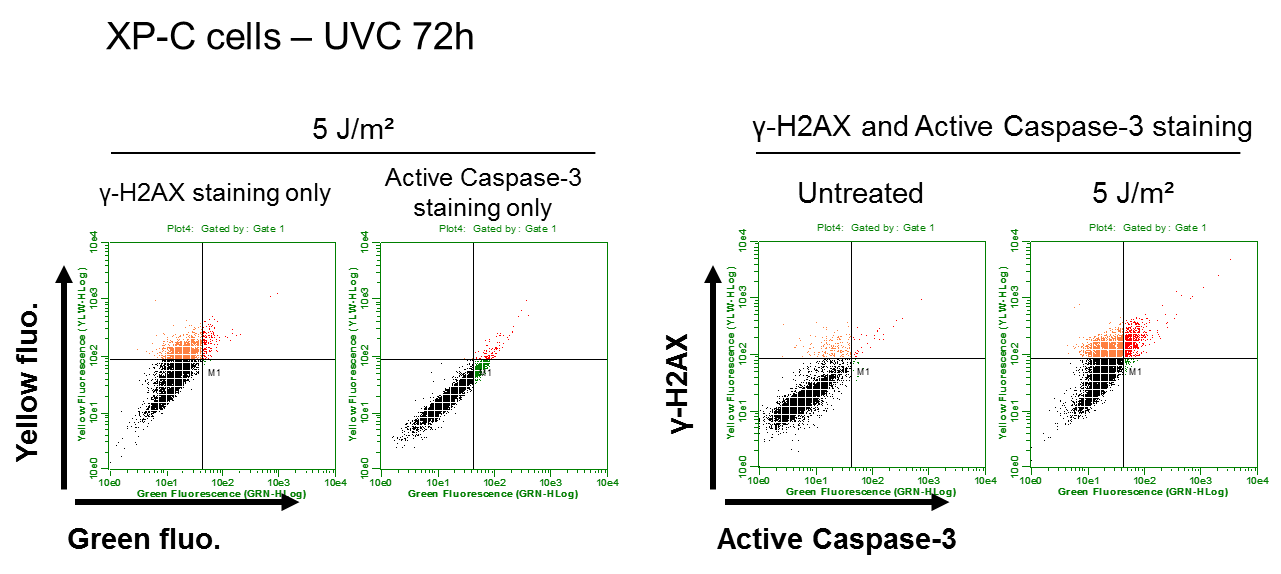


Figure S1: γ-H2AX (yellow fluorescence) and active Caspase-3 (green fluorescence) analyses by flow cytometry using XP-C cells 72 h after UVC exposure.
